# Multi-scale coupled attention for visual object detection

**DOI:** 10.1038/s41598-024-60897-8

**Published:** 2024-05-16

**Authors:** Fei Li, Hongping Yan, Linsu Shi

**Affiliations:** 1 China Tower Corporation Limited, No.9 Dongran North Street, Beijing, 100195 China; 2 China University of Geosciences, Xueyuan Road 29, Beijing, 100083 China

**Keywords:** Attention mechanism, Deep neural networks, Object detection, Self-attention learning, Transformer, YOLO, Attention, Intelligence, Electrical and electronic engineering

## Abstract

The application of deep neural network has achieved remarkable success in object detection. However, the network structures should be still evolved consistently and tuned finely to acquire better performance. This gears to the continuous demands on high performance in those complex scenes, where multi-scale objects to be detected are located here and there. To this end, this paper proposes a network structure called Multi-Scale Coupled Attention (MSCA) under the framework of self-attention learning with methodologies of importance assessment. Architecturally, it consists of a Multi-Scale Coupled Channel Attention (MSCCA) module, and a Multi-Scale Coupled Spatial Attention (MSCSA) module. Specifically, the MSCCA module is developed to achieve the goal of self-attention learning linearly on the multi-scale channels. In parallel, the MSCSA module is constructed to achieve this goal nonlinearly on the multi-scale spatial grids. The MSCCA and MSSCA modules can be connected together into a sequence, which can be used as a plugin to develop end-to-end learning models for object detection. Finally, our proposed network is compared on two public datasets with 13 classical or state-of-the-art models, including the Faster R-CNN, Cascade R-CNN, RetinaNet, SSD, PP-YOLO, YOLO v3, YOLO v5, YOLO v7, YOLOX, DETR, conditional DETR, UP-DETR and FP-DETR. Comparative experimental results with numerical scores, the ablation study, and the performance behaviour all demonstrate the effectiveness of our proposed model.

## Introduction

Object detection is one of the core issues in the field of computer vision, which has been extensively researched for a few decades. The main task is to identify all of the interested objects in images and determine their positions and categories. Due to the various appearances, postures, sizes, occlusions along with different lighting conditions, object detection has persistently been a challenging problem in computer vision.

Early detection algorithms mostly consist of two phases. The first phase attempts to detect a series of candidate regions for specific objects in an image, and the second phase is to classify candidate regions into classes and mark them with bounding boxes. Early methods are largely developed on the algorithms including Viola-Jones^1,2^, Histogram of Oriented Gradient (HOG)^3^, and Deformable Part Models (DPMs)^4–6^, and so on. Technically, in this family, visual features are extracted according to various rules designed manually with the observations on the characteristics of the objects, or according to mathematic operations like Harr wavelets, Gabor wavelets, filter banks, correlation coefficients, and so on. In the second phase, traditional classifiers, including the k-nearest neighbors, support vector machine, adaboost, and neural networks, are employed to infer the categories of regions. However, the procedures of feature extraction and classifier design are separated from each other, resulting in the fact that the systems are incapable of data adaptability and task-driven enhancement.

The burst of Convolutional Neural Network (CNN) has brought a revolutionary breakthrough to visual understanding. Following the rapid advances of deep learning, extensive models have emerged for object detection, achieving better and better results. These models can be mainly classified into two categories: two-stage detection and one-stage detection^7^. In a two-stage detection model, candidate regions are detected through a deep neural network, and then the candidate regions are refined and classified as a certain class of target object. This strategy is somewhat like the early detection algorithms. Among the two-stage detection models, the classical ones contain R-CNN, Spatial Pyramind Pooling Convolutional Network (SPPNet ), Fast R-CNN, Faster R-CNN, and Feature Pyramid Networks (FPN)^8–13^. R-CNN is the first algorithm that successfully applies deep learning to object detection^8^. Besides these models, there are also some other variations such as Mask R-CNN, Region-based Fully Convolutional Network (R-FCN), Cascade R-CNN, Libra R-CNN, NAS-FPN, and DetectoRS^14–18^. Most of these two-stage detection models can achieve high accuracy, but exhibit a relatively low speed.

In one-stage models, detecting candidate regions is not necessarily taken as an extra stage. That is, the class probabilities and position coordinate of the object are directly computed through a deep neural network. In this family, representative methods mainly include DenseBox^19^, Single Shot multi box Detector (SSD)^20^, DSSD (Deconvolutional Single Shot Detector)^21^, Retina-Net^22^, CornerNet^23^, Fully Convolutional One Stage (FCOS)^24^, RepPoints^25^, CenterNet^26^ and YOLO series^27–35^. Granted the fact that the detection rates may not be competent to those obtained from two-stage detection models, these one-stage models often possess a rather satisfying detection speed, which can be employed in real-time detection scenarios.

In parallel, Visual Transformers (ViTs)^36^ have been employed in object detection. Architecturally, transformer adopts a simple network structure, which relies only on the mechanism of attention^37^. By taking the ViT as its backbone, DEtection TRansformer (DETR) is the first model that applies transformer to the field of object detection^38^. Later, Deformable DETR was proposed to overcome the problem of slow convergence and limited feature spatial resolution in DETR^39^. Meanwhile, there are also some research works that follow the architecture of DETR, like Conditional DETR, UP-DETR, FP-DETR, and Group DETR^40–43^. Transformer-based detectors can achieve the state-of-the-art precisions. However, they need a huge number of epoches and a huge amount of computation memory to train the models well. In addition, Transformers are sensitive to hyper-parameters, leading to difficulties in convergence.

In contrast to the models in the two-stage family and those in the ViT-based family, models in the one-stage family are popularly applied in industrial scenarios. This is largely due to the fact that these models have light architectures, which are easier to be trained well. In addition, their inference speeds are relatively faster. Typically, the YOLO series, which are first introduced by Joseph et al.^27^ and followed by themselves and other researchers, have played an essential role in the evolution of one-stage object detection methods. Up to the early month in this year, the YOLO-based detector has issued the eighth version (YOLO v8)^44^. Technically, YOLO series provide good balance between various network scales and computing resources. With the iterative upgrading of model maturity, inference speeds have been enhanced version by version. These aspects facilitate their applications in many tasks that require real-time responses and limited computing resources.

Although YOLO series have achieved good performances, they have still limitations on multi-scale object detection tasks in practice, typically in the case that there exist large and small objects simultaneously to be detected. Such situations are often encountered in scenes with many artificial objects belonging to the same class but with large size ratios. To this end, many proposals have been practiced, and most of them follow upon the proposals with multi-scale feature fusion. Architecturally, some YOLO versions, such as YOLO v4, YOLO v5, and YOLOX^30,31,45^, have rendered an explicit neck network, which is designed to support top-down and bottom-up feature fusion. In other words, the multi-scale feature fusion is performed bilaterally level-by-level. Given an image including multi-scale objects, however, humans perceive them by looking at the contents in the image and comparing them mutually with each other. That is, beyond in a computational way of fusing the abstract or semantic features level by level, humans compare objects with different sizes mutually against each other in way of attentions both on their spaces and on their visual contents.

The above observation motivates us to develop a network under the YOLO framework, which can allow the cross-scale interaction both at the filter channel (namely feature extractor) granularity and at the spatial grid granularity. In parallel to human perception with a glance at the image, the importance of the multi-scale features should be assessed simultaneously as a whole. In deep learning, importance assessment always associates to the attention computation. For examples, attention mechanism is one of the key issues in deep models^46,47^. Without the guidance of some supervised information, such a goal can be achieved via self-attention learning.

In this paper, we propose a Multi-Scale Coupled Attention (MSCA) network for object detection. Architecturally, the MSCA module is comprised of a group of operations, including a Multi-Scale Coupled Channel Attention (MSCCA) module, and a Multi-Scale Coupled Spatial Attention (MSCSA) module. Both of these two modules mix together the multi-scale features, and take them equally as a whole for self-attention learning. Technically, the MSCCA focuses on learning from the channels of the multi-scale feature maps, while the MSCSA places the emphasis on learning from the information on the spatial grid. The MSCCA and MSCSA can be connected in series to be a deep structure with multiple MSCAs, which can be embedded as a plugin module into the YOLO frameworks. A large amount of experiments have been conducted to validate our model, exhibiting its superiority over the state-of-the art methods.

**Figure 1:**
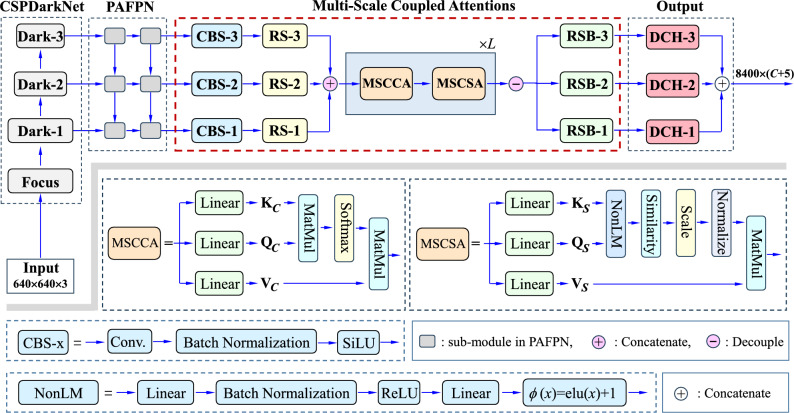
The main structure of our proposed Multi-Scale Coupled Attention network.

The main structure of our proposed Multi-Scale Coupled Attention network is illustrated in Fig. 1, which will be explicated in “Methods”. The main work and the contributions in this paper can be highlighted as follows.

- A Multi-Scale Coupled Attention (MSCA) network is developed for object detection. Accordingly, it consists of a Multi-Scale Coupled Channel Attention (MSCCA) module, and a Multi-Scale Coupled Spatial Attention (MSCSA) module. Both of these modules are developed under the framework of self-attention learning from the multi-scale feature maps. Acting as the neck network, the MSCA rectifies the multi-scale features without changing the formats of its inputs and outputs. This renders a different way from traditional proposals just for the goal of feature fusion in architecture design. As a result, it can be used as a plugin to enhance the performance of the existing models for object detection.
- Technically, the MSCCA is developed in terms of self-attention learning linearly on the channels. Due to the one-by-one relationship between the channels and the convolutional filters, the MSCCA measures actually the importance of multi-scale feature maps at the level of feature extractor. In parallel, the MSCSA is developed in terms of self-attention learning nonlinearly on the spatial grid by comparing the multi-scale features against each other. It captures the importance of spatial spaces of the multi-scale features. A new Non-Linear Mapping (NonLM) operation in the MSCSA is constructed to achieve this goal.
- The usability of the proposed MSCA network has been evaluated via extensive comparisons and rich ablation studies. More specifically, the advantages of our proposed network are well exhibited by comparison with other baseline networks. In addition, we have carefully designed the ablation study by gradually adding our contributions in different settings. The experimental results indicate that the MSCA network can help improve significantly the performance of the models in the YOLOX framework, demonstrating that our model can be widely used in industrial applications.

## Related works

### Detectors with multi-scale feature mapping

Granted the fact that a summary has been presented for some typical state-of-the-art object detection networks in “Introduction”, there are still some advanced and well-performed proposals for this issue, each of which provides a mechanism to utilize explicitly or implicitly the multi-scale features to achieve good performance.

Technically, Faster R-CNN^12^, SSD^20^, RetinaNet^22^ and FCOS^24^ are developed under anchor-based frameworks with different receptive fields, where features learned at previous scale are taken as the input to obtain the next scale. Differently, Cai et al.^48^ developed a Cascade R-CNN to guide the feature learning at different levels by multi-scale supervised information. It consists of a set of detectors with increasing values of Intersection over Union (IoU), which can gradually improve detection results. The detectors are trained stage by stage, with the latter detector utilizing the output of the previous one as its input to obtain higher quality predictions. Later, Sun et al. developed the Sparse R-CNN^49^, which is a sparse anchor-free framework for object detection. It rejects the dense concepts of anchor boxes or reference points, and starts directly from a sparse set of learned proposals without post-processing like the Non-Maximum Suppression (NMS) operations. The sparsity makes it possible to directly select a small number of object candidates from the multi-scale feature maps.

In the literature, Zhang et al. developed the Adaptive Training Sample Selection (ATSS)^50^ to bridge the gaps between anchor-based and anchor-free algorithms. According to the statistical characteristics of targets, the performance of those anchor-based and anchor-free detectors could be further improved by automatically selecting positive and negative samples including different scales of objects. In addition, You Only Look One-level Feature (YOLOF)^51^ is actually designed as an alternative FPN, which does not belong to the family of YOLO series. As is well known, FPN has made significant contributions to one-stage anchor-free object detection. From an optimization perspective, YOLOF introduces an alternative solution regardless of complex feature pyramids. In this framework, two central components are specified, namely, dilated encoder and uniform matching, which helps bring performance improvements.

In summary, how to utilize multi-scale features is one of the keys to improve the performance of the object detector. Different tricks have been employed for this issue in many classical models. However, few of them organize the multi-scale features as a whole by learning to measure their usability via cross-scale measurement for performance enhancement.

### The YOLO series

The YOLO series for object detection have been widely used in real-world applications. These models provide a good balance between network scales and computing resources. In contrast to the two-stage detection algorithms, the original YOLO^27^ directly predicts the coordinates of the bounding boxes of the objects and their categories. As the first version in this family, however, it performs poorly in handling small objects. Besides, it is easily influenced by the lighting changes.

YOLO v2^28^ introduces a new training with one dataset for position regression and another one for object classification, achieving faster prediction compared with the original YOLO. Later, YOLO v3^29^ employs the FPN to implement the feature fusions on three different scales. To improve the performance, it takes the Darknet-53 with residual links as its backbone^29^. In addition, binary cross entropy loss is adopted to tain the model. YOLO v4^30^ introduces the Cross Stage Partial Network (CSPNet)^52^ and the Darknet53 (together named as CSPDarknet^53^) to improve the accuracy, where a bottom-up feature pyramid is designed to achieve path aggregation of multi-scale features. Shortly later, YOLO v5^31^ is released with CIoU_Loss function^54^ and mosaic data augmentation tricks are used to improve the training speed and the accuracy.

There are also some more recently developed versions of YOLO series, like YOLOX, YOLO v6, YOLO v7, PP-YOLO, PP-YOLOE and YOLO v8^32–35,44,45^. In YOLO v6, both the backbone and the neck have been newly designed, and the decoupled head in YOLOX^45^ has been inherited with minor modifications. Besides, there are also improvements to the training strategy. YOLO v7 aims at various applications of CPUs and GPUs from edge devices to the cloud^33^, along with tricks of re-parameterizing and dynamic label assignment. PP-YOLO is derived from YOLO v3^34^ on the PaddlePaddle platform with neural architecture search. In parallel, PP-YOLOE^35^ renders an anchor-free network in the YOLO families with tricks of task alignment learning and task-aligned head to improve the performance and the processing speed.

Typically, among the YOLO series, YOLOX^45^ is a classic model, which is widely used in industrial application with different devices. It is constructed on YOLO v3 and YOLO v5, with the effective employment of the CSPDarkNet, the Path Aggregation FPN (PAFPN) and the SiLU activation layer^45^. Technically, it has the advantages in multi-scale feature fusion, excellent real-time detection speed, high detection accuracy, and unique decoupling head tasks.

In summary, the YOLO series have offered powerful ability of feature representations by combining the excellent modules along the pipeline of backbone, neck, and head. The multi-scale features are extracted via the hierarchical structure with multiple path aggregation. The feature aggregation is performed gradually level-by-level with top-down or/and bottom-up directions. However, none of the existing YOLO frameworks consider the multi-scale features as a whole to measure their usability mutually for object detection.

## Methods

### The main architecture and the motivation

Figure 1 illustrates the hierarchical structure of our proposed network. The network mainly consists of four parts: CSPDarkNet, PAFPN, Multi-Scale Coupled Attentions (MSCA), and the output layer. It is developed on the YOLOX framework. The CSPDarkNet, PAFPN, and the output layer are the standard units. Differently, the MSCA acts as the neck, which is the newly-designed module to achieve the feature mapping via multi-scale coupled attention.

For clarity, CSPDarkNet generates three scales of feature mappings for the next steps of network construction, where each Dark-x (x = 1, 2, 3) is a unit used in DarkNet53^29^. Before Dark-x, a focus module is introduced to slice the input image, in which the slices are concatenated together to reduce the number of the parameters and thus enhance the inference speed. Another basic module is the PAFPN, which provides a two-way fusion of the three-scale features respectively with the FPN module and the Path Aggregation (PA) module. In the FPN module, high-level feature information is transferred and fused to obtain a predicted feature map through up-sampling from top to bottom. In the PA module, the down-sampled small-scale feature map is integrated together with the large size feature map from bottom to top.

As demonstrated in Fig. 1, our MSCA takes the output of the CBS module as its input. It includes the Convolution (C), Batch Normalization (B) and SiLu activation (S). The batch normalization ensures the consistent distribution between the output of each layer and the input of the subsequent layer, which can make the model more stable during training. Compared to the activation function ReLU, the SiLU function has exhibited stronger nonlinear characteristics. Actually, it can help solve the problem of gradient dispersion in the case that the weighted sum is less than zero. At the same time, it has inherited the advantage of faster convergence of the ReLU. Besides the above advantages, in our design, the three CBS modules are employed to align the feature maps into the same dimensionality to achieve the goal of mixing the multi-scale features for joint learning.

In Fig. 1, like the CSPDarkNet, the PAFPN also keeps the output with three-scales of feature maps. In the YOLOX framework, each feature map generated by the PAFPN module within a single scale is then treated independently, without any interaction between different scales. This yields a computational pipeline for the final object detection via the output layer in Fig. 1. However, for multi-scale objects, humans attempt to understand them by combining them together and taking attentions on their visual appearances as well as their shape sizes. This observation motivates us here to mix together all of the different scales of feature maps, without fusing them as most traditional way like level-by-level or scale-by-scale. Naturally, the YOLOX framework with the PAFPN module gears to the need for taking the multi-scales of feature maps as the explicit input, where such a feature mixture could be performed in a computational way.

Following the above motivation, self-attention learning will be developed on the mixture of the multi-scale feature maps. In Fig. 1, the Multi-Scale Coupled Attentions (MSCA) network will be constructed to achieve this goal. It includes a Multi-Scale Coupled Channel Attention (MSCCA) module and a Multi-Scale Coupled Spatial Attention (MSCSA) module. With tricks of self-measurement, the MSCCA is to evaluate the importance of the multi-scale features within the filter channel granularity, while the MSCSA is to assess the importance in the spatial gird granularity.

**Figure 2:**
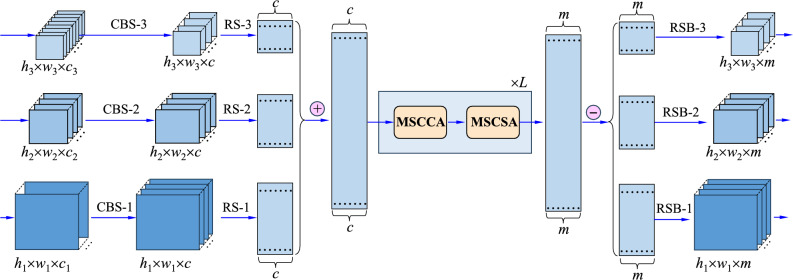
The data streams with inputs and outputs for the important modules in the MSCA.

Architecturally, the MSCA will be repeated *L* times, which will be used as the neck part in the whole network. Finally, by separating its output back in a scale decoupling way, multi-scale features have been rectified as a whole, which will be finally delivered to the output layer for final category estimation and position regression within the minimum loss for the task of object detection. For clarity, Fig. 2 shows the data streams with inputs and outputs for the important modules in the MSCA.

### The MSCCA

The task of the MSCCA sub-module is to evaluate the importance of the multi-scale features in view of channel attention. This goal will be achieved linearly along the attention on channels.

As demonstrated in Fig. 1, it takes the outputs of the three CBS modules as its input. Accordingly, we suppose that the three-scale feature maps generated by the PAFPN are recorded respectively by tensors $\textbf{P}_1$, $\textbf{P}_2$ and $\textbf{P}_3$. More formally, each tensor $\textbf{P}_i$ (*i*=1, 2, 3) is formated in ${{\mathbb {R}}}^{w_{i}\times h_{i} \times c_{i}}$, where $w_{i}$ and $h_{i}$ are the width and height of the *i*-th feature map, and $c_{i}$ is the number of channels. With scale changing, the size of the feature map reduces half at each scale, namely $w_{2}=w_{1}/2$, $h_{2}=h_{1}/2$, and $w_{3}=w_{2}/2$, $h_{3}=h_{2}/2$. Note that, to achieve a powerful ability of abstract representation learning, usually we have $c_1 \le c_2 \le c_3$. As the channel numbers are not equal to each other, the CBS module is used to align their channels as follows:

1 $$\begin{aligned} {\textbf{C}}_i = CBS_i \left( {\textbf{P}}_i \right) \; \in \; {{\mathbb {R}}}^{w_i \times h_i \times c},\quad i = 1,2,3, \end{aligned}$$

where $CBS_i(\cdot )$ corresponds to the module CBS-*i* in Fig. 1, ${\textbf{C}}_i$ is the output tensor of the module CBS-*i*, and *c* is the number of channels for all three scales. It can be seen that the spatial size in each scale of feature map will be kept unchanged, while the number of the channel keeps the same for all feature maps. This treatment offers us to develop a mechanism for learning from them as a whole.

Note that the three tensors ${\textbf{C}}_i$ (*i* = 1, 2, 3) at different scales have different tensor sizes. To mix them together and achieve the goal of the MSCCA, we further reshape each of them into a two-dimensional matrix. To this end, a dependent RS (reshape) module is introduced to pick up the features in *c* channels pixel by pixel. Formally, the RS module fulfills the following operation:

2 $$\begin{aligned} {\textbf{X}}_i = RS_i \left( {\textbf{C}}_i \right) \; \in \; {{\mathbb {R}}}^{s_i \times c}, \quad i = 1,2,3. \end{aligned}$$

where $RS_i(\cdot )$ associates to the module RS-*i* in Fig. 1, ${\textbf{X}}_i$ is the output matrix of the module CBS-*i*, and $s_i = w_i \times h_i$.

Now, the multi-scale features in ${\textbf{X}}_i$ (*i* = 1, 2, 3) are mixed together into a large matrix $\textbf{X}$. Then, it turns out that

3 $$\begin{aligned} {\textbf{X}} = \left[ {{\textbf{X}}_1^T,{\textbf{X}}_2^T,{\textbf{X}}_3^T } \right] ^T \in \; {{\mathbb {R}}}^{s \times c}, \end{aligned}$$

where $s=s_1+s_2+s_3$, and the superscript *T* stands for the transposition operation of matrix. For clarity, Fig. 2 illustrates the above data re-organization.

In Eq. (3), ${\textbf{X}}$ collects all of the features with different scales. With this form, all of them will be equally treated later to learn the attentions. Now we introduce the linear self-attention to evaluate the importance of the multi-scale features. To this end, the self-attention measurement is expressed as a dot-product in a latent linear space. Then, totally three groups of linear projections are learned from the input $\textbf{X}$:

4 $$\begin{aligned} \textbf{Q}_{C}=\textbf{X} \times \textbf{W}_{qC}, \end{aligned}$$

5 $$\begin{aligned} \textbf{K}_{C}=\textbf{X} \times \textbf{W}_{kC}, \end{aligned}$$

6 $$\begin{aligned} \textbf{V}_{C}=\textbf{X} \times \textbf{W}_{vC}, \end{aligned}$$

where $\textbf{Q}_{C} \in {\mathbb {R}}^{s \times d}$, $\textbf{K}_{C} \in {\mathbb {R}}^{s \times d}$, $\textbf{V}_{C} \in {\mathbb {R}}^{s \times d}$ are three two-dimensional matrices, which are recorded as the queries, keys and values learned linearly respectively from $\textbf{X}$, for convenience. In the above equations, $\textbf{W}_{qC} \in {\mathbb {R}}^{c \times d}$, $\textbf{W}_{kC} \in {\mathbb {R}}^{c \times d}$, $\textbf{W}_{vC} \in {\mathbb {R}}^{c \times d}$ are the linear projection matrices (namely the filters) to be learned from data, where *d* is the dimensionality of the projected linear space. In addition, the subscript “C” associates to “channel”, and “$\times$” stands for matrix multiplication.

Furthermore, the queries in $\textbf{Q}_{C}$ and the keys in $\textbf{K}_{C}$ will be employed to perform the similarity measurement at the channel granularity. According to the proposal in^47^, which is introduced to reduce the computational complexity when performing the self-attention, the cross-covariance attention is calculated on the queries and keys. Accordingly, we have

7 $$\begin{aligned} {\textbf{C}}_{attention} = \mathrm{{softmax}}\left( {{{{\bar{\textbf{Q}}}_C^T \times {\bar{\textbf{K}}}_C } / {\sqrt{d} }}} \right) , \end{aligned}$$

where ${\textbf{C}}_{attention} \in {\mathbb {R}}^{d \times d}$ records the cross-covariance attention, ${\bar{\textbf{Q}}}_C$ and ${\bar{\textbf{K}}}_C$ are the normalized matrices of ${\textbf{Q}}_C$ and ${\textbf{K}}_C$ respectively, and “$\times$” and softmax($\cdot$) stand for the “MatMul” and the “Softmax” operators respectively in Fig. 1 (see the bottom-left panel of MSCCA). Here, ${\bar{\textbf{Q}}}_C$ and ${\bar{\textbf{K}}}_C$ are obtained by dividing the L2-norm of each column vector in the matrix. According to the suggestion given in^47^, such a treatment can help confine the entities of the cross-covariance matrix ${\bar{\textbf{Q}}}_C^T \times {\bar{\textbf{K}}}_C$ into the interval $[-1,1]$. This will yield stationary attentions for model training. Furthermore, with the softmax($\cdot$), the importance of cross-scale features are mapped into [0, 1].

Finally, with the normalized cross-covariance attention, the result with the coupled channel attention can be obtained from the values in $\textbf{V}_{C}$. Formally, we have

8 $$\begin{aligned} {\textbf{Y}}\mathrm{{ = }}{\textbf{V}}_{\mathrm{{C}}} \times {\textbf{C}}_{attention}, \end{aligned}$$

where ${\textbf{Y}}$ records the final output of the MSCCA module.

As can been seen from Eq. (8), ${\textbf{Y}} \in \mathbb R^{s \times d}$ yields a convex combination of values along the channel dimensionality, taking all of the *s* samples obtained in Eq. (3) at different scales as equal partners. Computationally, such a self-attention is originated from the channels or filters. This fact indicates that the MSCCA module is to extract the significant channels of the feature maps. As filters do actually act as feature extractor, this treatment gears to human vision perception via feature importance on the visual appearances of objects.

### The MSCSA

The task of the MSCSA module is to evaluate the importance of the multi-scale features in view of spatial attention. Technically, this goal will be achieved along the nonlinear Transformer via cross-Gram attention on spatial grids with different scales.

As demonstrated in Fig. 1, it takes the output of the MSCCA module as its input. Like the operations in the MSCCA module, the linear Transformer with self-attention is also introduced to assess the importance of the multi-scale features at the level of spatial grid. Correspondingly, three groups of linear projections are then learned from the input $\textbf{Y}$:

9 $$\begin{aligned} \textbf{Q}_{S}=\textbf{Y} \times \textbf{W}_{qS}, \end{aligned}$$

10 $$\begin{aligned} \textbf{K}_{S}=\textbf{Y} \times \textbf{W}_{kS}, \end{aligned}$$

11 $$\begin{aligned} \textbf{V}_{S}=\textbf{Y} \times \textbf{W}_{vS}, \end{aligned}$$

where $\textbf{Q}_{S} \in {\mathbb {R}}^{s \times m}$, $\textbf{K}_{S} \in {\mathbb {R}}^{s \times m }$, $\textbf{V}_{S} \in {\mathbb {R}}^{s \times m}$ are three two-dimensional matrices, which are recorded as the queries, keys and values learned linearly respectively from $\textbf{Y}$, for convenience. In the above equations, $\textbf{W}_{qS} \in {\mathbb {R}}^{d \times m}$, $\textbf{W}_{kS} \in {\mathbb {R}}^{d\times m}$, $\textbf{W}_{vS} \in {\mathbb {R}}^{d \times m}$ are the linear projection matrices to be learned from data, where *m* is the dimensionality of the projected linear space. In addition, the subscript “S” associates to “Spatial grid”, and “$\times$” stands for matrix multiplication.

Now it seems that it is a natural way to get the values along the operations fulfilled by the MSCCA module. But differently, beyond learning the self-attention from the cross-covariance matrix $\textbf{Q}_{S}^T \times \textbf{K}_{S} \in {{\mathbb {R}}}^{m \times m}$ , now we need to consider the cross-Gram matrix $\textbf{Q}_{S} \times \textbf{K}_{S}^T \in {{\mathbb {R}}}^{s \times s}$ for determining the spatial attentions. Note that the cross-Gram matrix actually records the similarities between the queries in $\textbf{Q}_{S}$ and the keys in $\textbf{K}_{S}$. This motivates us to calculate the similarity in a latent feature space. In view of kernel learning^55^, the similarity between two points will be calculated in a reproducing kernel Hilbert space, which is spined out from a nonlinear mapping. In such a Hilbert space, the similarity can be calculated via a kernel function. However, classic kernel functions such as Gaussian functions and polynomials have fixed forms with super-parameters, which could limit the representation power of the deep model. Thus, alternatively, we develop a nonlinear mapping to achieve this goal.

The Non-Linear Mapping (NonLM) is actually a module of neural network. As demonstrated in the bottom left panel in Fig. 1, it is unfolded as a series of transformations including a layer of fully-connected forward layer (Linear), a layer of Batch Normalization (BN), a ReLU layer, a layer of fully-connected forward layer (linear), and an activation function with form $\phi (x)$. More specifically, we denote ${\textbf{Q}}_S = [{\textbf{q}}_1^T ,{\textbf{q}}_2^T , \ldots ,{\textbf{q}}_s^T ]^T$ and ${\textbf{K}}_S = [{\textbf{k}}_1^T ,{\textbf{k}}_2^T , \ldots ,{\textbf{k}}_s^T ]^T$, where ${\textbf{q}}_i \in {{\mathbb {R}}}^m$ (*i*=1, 2, $\ldots , s$) and ${\textbf{k}}_j \in {{\mathbb {R}}}^m$ (*j*=1, 2, $\ldots , s$) are the *i*-th row vector of ${\textbf{Q}}_S$ and the *j*-th row vector of ${\textbf{K}}_S$, respectively. For clarity, let ${\mathcal {D}}$ collect the set of all these 2*s* vectors, namely ${\mathcal {D}} = \{ {\textbf{q}}_1 ,{\textbf{q}}_2 , \ldots ,{\textbf{q}}_s, {\textbf{k}}_1 ,{\textbf{k}}_2 , \ldots ,{\textbf{q}}_s \}$. For each vector *x* in ${\mathcal {D}}$, the NonLM module maps it as follows:

12 $$\begin{aligned} {\hat{{\textbf{x}}}} = NonLM(\textbf{x}) = \phi \left( {f_2 \left( {\mathrm{{ReLU}}\left( {\mathrm{{BN}}\left( {f_1 \left( {\textbf{x}} \right) } \right) } \right) } \right) } \right) , \forall \; \textbf{x} \; \in {\mathcal {D}}, \end{aligned}$$

where ${\hat{{\textbf{x}}}}$ records the output of the NonLM module, $\phi (x) = elu(x)+1$, in which *elu*(*x*) is an exponential linear unit^46,56^, and $f_1(\cdot )$ and $f_2(\cdot )$ correspond to the first and the second Linear layers with *m* nodes in the NonLM module. In Eq. (12), ReLU stands for the rectified linear unit, and BN is the batch normalization. It is worthy pointing out that BN is performed not on a single point *x* but on a subset of mini-batch samples in the training work setting.

The above NonLM module provides a function that generalizes a common mapping for similarity measurement. Based on Eq. (11), now for the *i*-th row vector ${\textbf{q}}_i$ in ${\textbf{Q}}_S$ and the *j*-th row vector ${\textbf{k}}_j$ in ${\textbf{K}}_S$, their similarity can be calculated in a latent feature space as follows:

13 $$\begin{aligned} \begin{aligned} sim\left( {{\textbf{q}}_i,{\textbf{k}}_j } \right) = {\hat{{\textbf{q}}}}_i^T \,{\hat{{\textbf{k}}}}_j =&\left( {NonLM\left( {{\textbf{q}}_i } \right) } \right) ^T \left( {NonLM\left( {{\textbf{k}}_j } \right) } \right) , \\&\quad i, j = 1, 2, \ldots , s, \end{aligned} \end{aligned}$$

where $sim\left( {{\textbf{q}}_i,{\textbf{k}}_j } \right)$ stands for the similarity between ${\textbf{q}}_i$ and ${\textbf{k}}_j$, which are calculated in the *m*-dimensional latent feature space.

Finally, we can perform the self-attention operation on the values in ${\textbf{V}}_S$. For clarity, we denote ${\textbf{V}}_S = [{\textbf{v}}_1^T ,{\textbf{v}}_2^T , \ldots ,{\textbf{v}}_s^T ]^T$, where ${\textbf{v}}_i \in {{\mathbb {R}}}^m$ (*i*=1, 2, $\ldots , s$) is the *i*-th row vector of ${\textbf{V}}_S$. Formally, for each ${\textbf{v}}_i$, we have

14 $$\begin{aligned} {\hat{{\textbf{v}}}}_i = \frac{{\sum \limits _{j = 1}^s {sim\left( {{\textbf{q}}_i,{\textbf{k}}_j } \right) {\textbf{v}}_j } }}{{\sum \limits _{n = 1}^s {sim\left( {{\textbf{q}}_i,{\textbf{k}}_n } \right) } }}, \quad i = 1, 2, \ldots , s, \end{aligned}$$

${\hat{{\textbf{v}}}}_i \in {{\mathbb {R}}}^m$ is the result mapped by the self-attention operation. The sum in the denominator is a normalization factor. In Eq. (14), ${\hat{{\textbf{v}}}}_i$ is actually a convex combination of values among all of the *s* features spatially with different scales. Methodologically, this treatment achieves our goal of mixing multi-scale features together and measuring them mutually to enhance the performance, which gears to human vision perception via measuring their sizes of objects as a whole on the image grid.

Furthermore, the output of our designed MSCSA module can be organized as a matrix by collecting all of the vectors ${\hat{{\textbf{v}}}}_i$ (*i*=1, 2, $\ldots , s$) together:

15 $$\begin{aligned} {\hat{{\textbf{Z}}}} = \left[ {{\hat{{\textbf{Z}}}}_1^T \;|| \; {\hat{{\textbf{Z}}}}_2^T \; || \; {\hat{{\textbf{Z}}}}_3^T } \right] ^T \in {{\mathbb {R}}}^{s \times m}. \end{aligned}$$

where ${{\hat{\textbf{Z}}}}$ collects all of the mapped results, “||” separates the three scales from each other, and ${\hat{{\textbf{Z}}}}_1$, ${\hat{{\textbf{Z}}}}_2$ and ${\hat{{\textbf{Z}}}}_3$ record the results corresponding the three scales. For clarity, they have the following forms:

16 $$\begin{aligned} {\hat{{\textbf{Z}}}}_1= & {} \left[ {{\hat{{\textbf{v}}}}_1^T,{\hat{{\textbf{v}}}}_2^T, \ldots ,{\hat{{\textbf{v}}}}_{s_1 }^T } \right] ^T \in {{\mathbb {R}}}^{s_1 \times m}, \end{aligned}$$

17 $$\begin{aligned} {\hat{{\textbf{Z}}}}_2= & {} \left[ {{\hat{{\textbf{v}}}}_{s_1 + 1}^T,{\hat{{\textbf{v}}}}_{s_1 + 2}^T, \ldots ,{\hat{{\textbf{v}}}}_{s_1 + s_2 }^T } \right] ^T \in {{\mathbb {R}}}^{s_2 \times m}, \end{aligned}$$

18 $$\begin{aligned} {\hat{{\textbf{Z}}}}_3= & {} \left[ {{\hat{{\textbf{v}}}}_{s_1 + s_2 + 1}^T,{\hat{{\textbf{v}}}}_{s_1 + s_2 + 2}^T, \ldots ,{\hat{{\textbf{v}}}}_s^T } \right] ^T \in {{\mathbb {R}}}^{s_3 \times m}. \end{aligned}$$

Finally, the features are decoupled scale by scale from matrix ${{\hat{\textbf{Z}}}}$. That is, we re-shape each matric back to be a three-dimensional tensor via the operation “RSB” in Fig. 1. Then, it follows

19 $$\begin{aligned} {\textbf{Z}}_1 = RSB\left( {{\hat{{\textbf{Z}}}}_1 } \right) ,\;\; {\textbf{Z}}_2 = RSB\left( {{\hat{{\textbf{Z}}}}_2 } \right) , \;\; {\textbf{Z}}_3 = RSB\left( {{\hat{{\textbf{Z}}}}_3 } \right) . \end{aligned}$$

In Eq. (19), $RSB(\cdot )$ stands for the “re-shape back” operation. In addition, ${\textbf{Z}}_1 \in R^{w_1 \times h_1 \times m }$, ${\textbf{Z}}_2 \in R^{w_2 \times h_2 \times m }$, and ${\textbf{Z}}_3 \in R^{w_3 \times h_3 \times m }$ are three tensors, which are taken as the outputs of the operators RSB-1, RSB-2, and RSB-3, respectively.

### The configurations of the model

**Figure 3:**
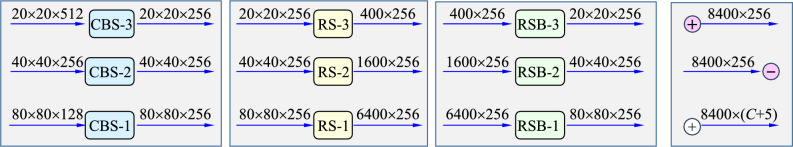
The parameter configurations in one MSCA module.

As mentioned in “The main architecture and the motivation” and demonstrated in Fig. 1, the whole model includes the CSPDarkNet, PAFPN, MSCA, and the output layer. The CSPDarkNet and the PAFPN are taken jointly as the backbone, which are designed as standard structures in YOLOX^45^. Meanwhile, the MSCA is employed as the neck network, which is designed in this work to enhance the performance. With the detailed design in “The MSCCA and The MSCSA”, the MSCA is a sequence of modules MSCCA and MSCSA with a length equal to *L*. Methodologically, our MSCA module supports any size of feature maps in (1) as its input, and any size of features in Eq. (19) as its output. In practice, the size of the deep model is actually limited by the computation resources and the scale of training data. Comprehensively, at first, the size is set as $640 \times 640 \times 3$ for RGB images. Along this setting, Fig. 3 illustrates the parameter configurations for the MSCA network. That is, in “The MSCCA”, we have $w_1 = h_1 = 80$, $w_2 = w_2 = 40$, and $w_3 = h_3 = 20$, and $c_1 = 128$, $c_2 = 256$, and $c_3 = 512$. Note that, the dimensions *c* in Eq. (2), *d* in Eqs. (4)–(6), and *m* in Eqs. (9)–(10) are all set to be 256 in our model. Thus, it is seen that the modules CBS-1, CBS-2, and CBS-3 align the input tensors with different dimensions of features into a unified feature space for performing the operations in the MSCA.

Beside the topological configuration to build up the model structure, the loss function is also a very important configuration to associate the learning task and the training effectiveness and efficiency. To this end, we use the Complete-IoU (CIoU) loss^57^ to train the model for position regression, which has the following form:

20 $$\begin{aligned} \begin{aligned} {\mathcal {L}}_{CIoU}=1-IoU+\frac{dist^{2}\left( {\textbf{b}}, {\textbf{b}}^{g t}\right) }{c^{2}}+\alpha v. \end{aligned} \end{aligned}$$

In Eq. (20), *IoU* is the intersection area of the ground truth box and the detected bounding box divided by their union, ${\textbf{b}}$ is the central point of the detected bounding box, and ${\textbf{b}}^{g t}$ is the central point of the ground truth box, $dist\left( \cdot , \cdot \right)$ stands for the distance between the two central points, *c* is the diagonal length of the smallest enclosing box covering the two boxes, *v* measures the consistency of aspect ratio, which is determined by the statistic values of the sizes of the objects, and $\alpha$ is automatically given according to *v*. All these parameters are determined as the suggestion in^57^. In addition, for the task of category classification, the traditional loss function of cross entropy is employed to fulfill this goal.

## Results

### Datasets

In this work, two public challenging datasets have been employed to evaluate the performance of our model. One is the well known COCO dataset, and the other is the KITTI dataset^58,59^.

#### The COCO dataset

**Figure 4:**
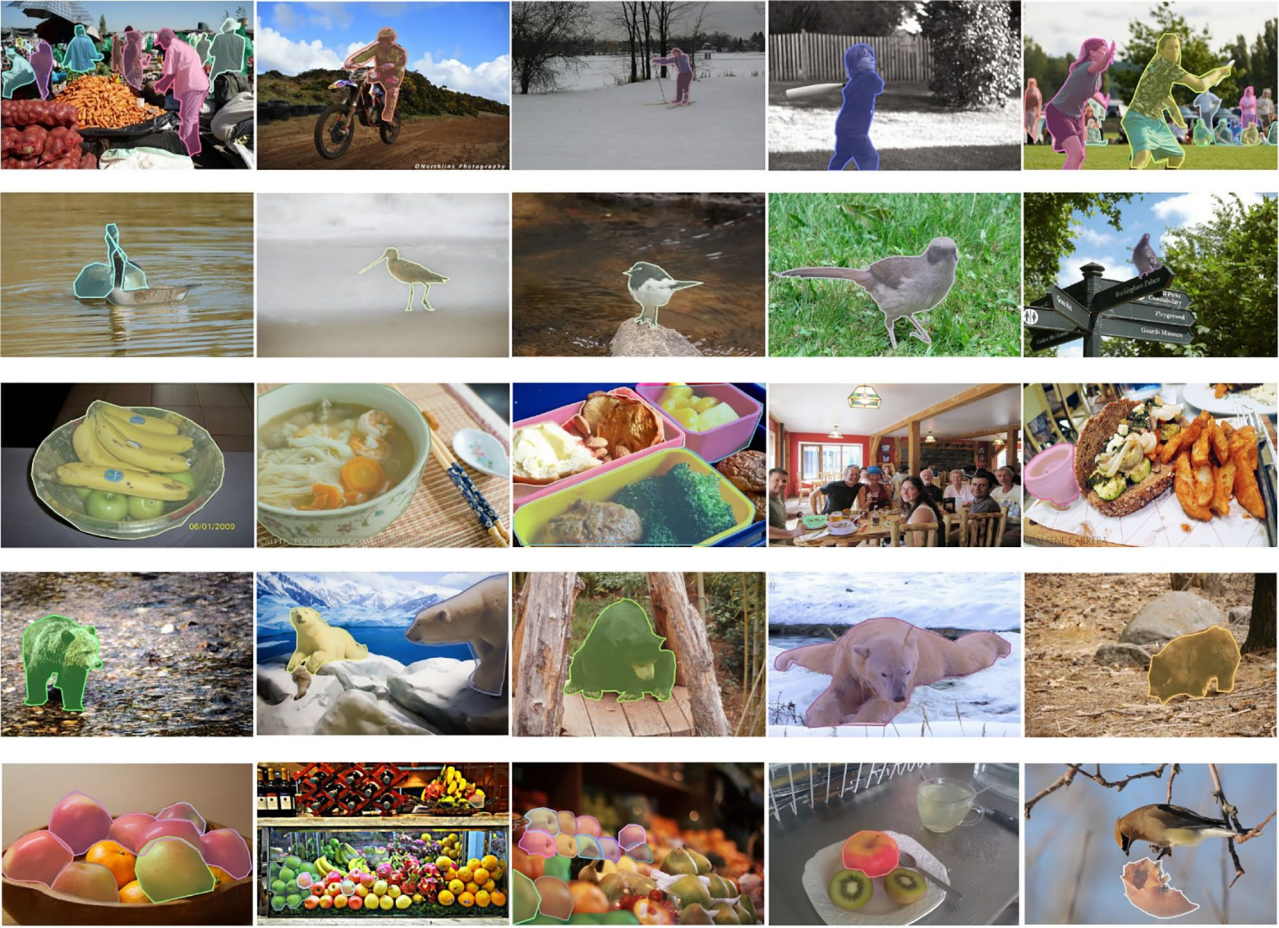
Some examples of annotated images in the COCO dataset, with labels Person, Bird, Bowl, Bear and Apple.

The COCO dataset is a large-scale benchmark. It consists of more than 330K images, among which 220K images are well labeled, and the labels of the rest 110K images have not been published by the authors. More specifically, there are about 1.5 million targets in this dataset, including 80 target categories (e.g., pedestrians, cars, elephants, etc.) and 91 stuff categories (e.g., grass, walls, sky, etc.). The COCO dataset is initially developed for image segmentation, and now has been widely-used in object detection, dense pose estimation, key-points detection, stuff segmentation, panoptic segmentation, and image captioning. Typically, annotations for object detection can be fulfilled automatically by marking the object regions as rectangle bounding boxes. Figure 4 demonstrates five categories of annotated sample images for examples, labeled as Person, Bird, Bowl, Bear and Apple.

In the experiments, the images of 80 target categories are employed to train our model and assess its performance. Specifically, the training subset consists of 118K images. Each image contains seven categories of objects on average, where the largest number of objects in one image is 63. The model is validated on 5K images. It is worthy pointing out that such a division of the images in COCO dataset is given in advance, which is now popularly-used for object detection in the field of computer vision.

#### The KITTI dataset

**Figure 5:**
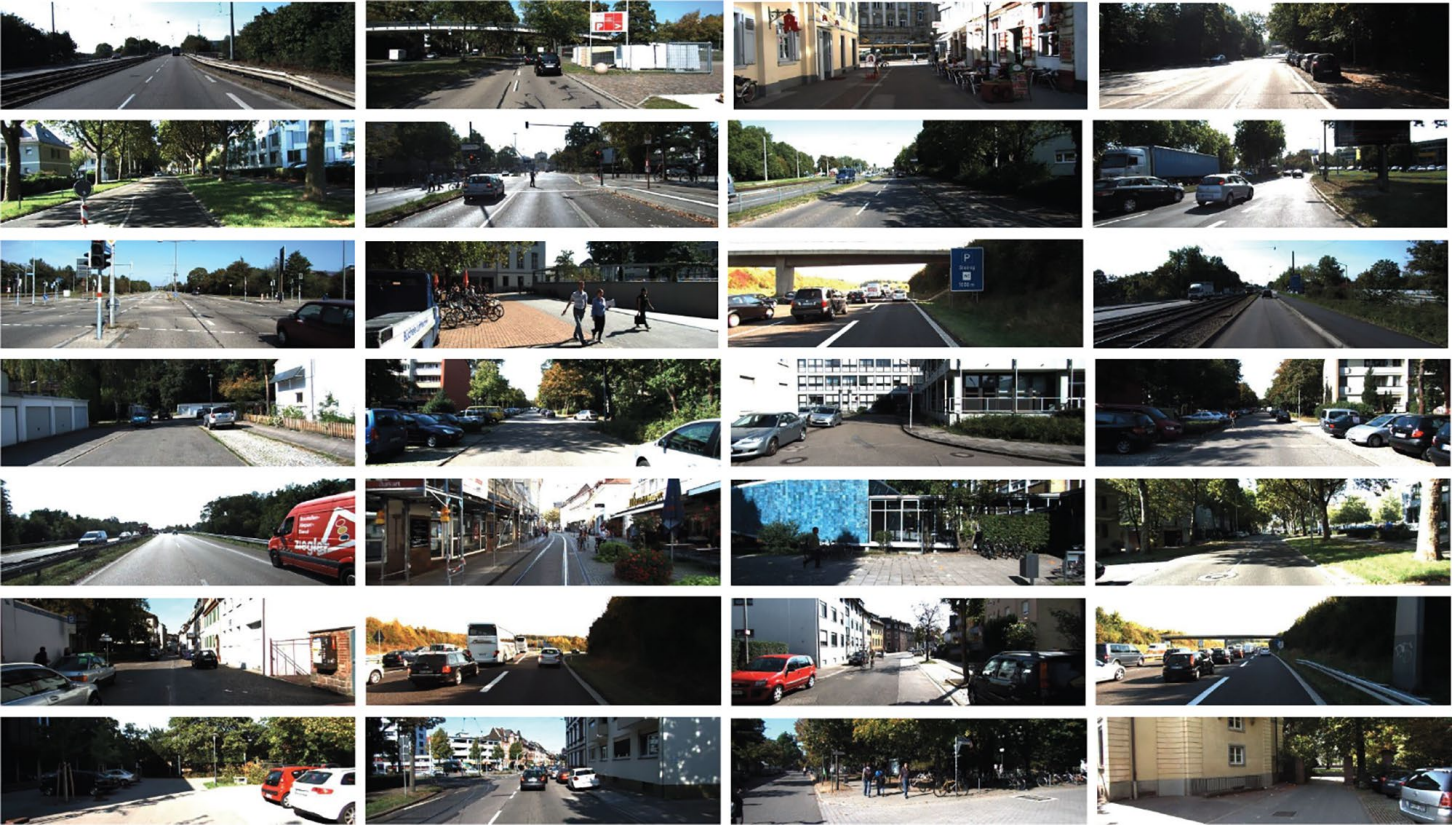
Some examples in the KITTI dataset, with labels Pedestrian, Car, Van, Truck, and Cyclist.

The KITTI dataset is currently the largest dataset for evaluating algorithms developed in the automatic driving scenarios. It is used to evaluate the performance of vision technologies such as stereo, optical flow, visual odometry, object detection (including 2D, 3D and aerival-view), and tracking in automotive environments. This dataset contains real images collected from urban, rural, and highway scenes, with up to 15 vehicles and 30 pedestrians in each image, as well as varying degrees of occlusion and truncation. Totally, it consists of color images (12GB), point clouds (29GB), and label data (5MB). The subset for object detection consists of 7481 training images, and 7518 test images. The dataset includes a total of 80,256 labeled objects, which belong to eight classes, namely, Car, Van, Truck, Tram, Pedestrian, Person (sitting), Cyclist, and Misc. Some examples of KITTI images are presented in Fig. 5, including five categories of objects belonging to Pedestrian, Car, Van, Truck, and Cyclist.

In this work, following the settings used in^60^, the two categories of Person and Pedestrian are merged together as a new Pedestrian category. Furthermore, the images in the five categories of Car, Van, Truck, Cyclist and Pedestrian are employed in our experiments. According to the experimental setting, all these images are divided into training subset, validation subset and test subset via 8:1:1 ratios, respectively. The models are trained on the training subset, and evaluated on the test subset.

### Comparison approaches

In our experiment, totally 13 classic models have been compared with our proposed model. Within the CNN frameworks, these models perform the tricks on multi-scale feature maps for fine object detection in different ways. For convenience, we summarize them briefly as follows:

- Faster R-CNN. A famous two-stage network for object detection^12^. It introduces the Region Proposal Network (RPN) to replace the selective search in the old versions of R-CNN, which is attached to the main structure to generate the candidate boxes. As a result, Faster R-CNN can greatly reduce the processing time while maintaining the detection accuracies.
- Cascade R-CNN. It belongs to the R-CNN family, which is an excellent two-stage model and widely applied to object detection. It has a cascade structure with a sequence of detectors, which is trained with stage by stage by increasing IoU thresholds.
- SSD. It is a classical one-stage multi-box object detector, where a multi-scale feature detection strategy is built up to maintain high inference accuracy^20^. In SSD, the bounding boxes are assigned discretely with different aspect ratios and scales at each location in feature map.
- RetinaNet. It is one-stage deep network for object detection^61^. Technically, it is a dense target detection network developed on multi-scale feature pyramid. Focal-loss weighted cross entropy function is introduced to suppress the loss from the negative samples.
- YOLO series. Here the four versions of YOLO v3^29^, YOLO v5^31^, YOLO v7^33^, and YOLOX^45^ are employed to compare with our model. These versions are popularly used in real-world applications.
- PP-YOLO. It is developed on YOLO v3 with the ResNet as its backbone^34^. The term “PP” stands for the platform PaddlePaddle. In PP-YOLO, various existing tricks, which do not increase the number of model parameters, are combined together to achieve the goal of accuracy enhancement.
- DETR and its variants. Here we employ DETR^38^ series as the baselines because they are typical transformer based methods, representing the advanced methods which can achieve SOTA nowadays. Besides DETR, its variants are conditional DETR^40^, UP-DETR^41^, and FP-DETR^42^.

### Experimental settings and evaluation metrics

In the experiments, the popularly-used Stochastic Gradient Descent (SGD) optimization algorithm is employed to train the proposed method. On the COCO dataset, for our method and the YOLOX, the batch size is taken as 64, and the total number of training epochs is set to be 300. On the KITTI dataset, the batch size is taken as 64, and the total number of training epochs is set to be 300. The learning rate $\eta$ is initially set as 0.01, the momentum is set to be 0.9, and the weight decay is taken as 0.004 during iterations. In the experiments, for the nine models described in “Comparison approaches”, the experiment settings as well as the important hyper-parameters being used during training are kept as those suggested by the authors in the original works.

During training, all the models adopt their corresponding pre-trained networks on the ImageNet classification dataset. On the COCO dataset, six evaluation metrics are adopted to evaluate the performance, including the mean Average Precision (mAP), Average Precision under the IoU equal to 0.75 (AP_75), Average Precision under the IOU = 0.50 (AP_50), Average Precision of small objects AP_s), Average Precision of medium objects (AP_m) and Average Precision of large objects (AP_l). On the KITTI dataset, following the work conducted by Jia et al.^62^, three evaluation metrics are adopted to evaluate the performance, including the Precision (P), Recall (R), and Average Precision under the IOU = 0.50 (AP_50).

More specifically, the metrics mAP, AP_50, AP_75, AP_s, AP_m and AP_l are popularly used in object detections, which are calculated from the scores of the IoU, AP, and Recall. Thus, they act as a set of comprehensive metrics to asses the goodness of the model. These scores are calculated as follows:

- Intersection over Union (IoU): IoU is defined as the area of overlap between the predicted bounding box and the ground truth bounding box, divided by the area of union of the two boxes. It is calculated as:
  21 $$\begin{aligned} \text {IoU} = \frac{\text {Area of the overlap}}{\text {Area of the union}}. \end{aligned}$$
- Precision: Precision is the ratio of the correctly predicted positive observations to the total predicted positives:
  22 $$\begin{aligned} Precision = \frac{\text {True Positives}}{\text { True Positives + False Positives}}. \end{aligned}$$
- Recall: Recall (Sensitivity) is the ratio of the correctly predicted positive observations to all of the observations in classes:
  23 $$\begin{aligned} \text {Recall} = \frac{\text {True Positives}}{\text {True Positives + False Negatives}}. \end{aligned}$$
- Average Precision (AP): Average precision measures the average precision value against the recall value over 0 to 1. Let *P*(*r*) be the precision at recall *r*, we have
  24 $$\begin{aligned} \text {AP} = \int _{0}^{1} P(r) dr. \end{aligned}$$
- Mean Average Precision (mAP): It averages the APs over a series of IoU thresholds such as those in [0.5, 0.95] with 0.05 increment:
  25 $$\begin{aligned} \text {mAP} = \frac{\sum _{t=1}^{T} AP_t}{T}. \end{aligned}$$
  where *T* is the total number of the thresholds.
- AP_50: It is an AP score in the case of IoU = 0.5. In other words, a predicted bounding box is considered to be a true positive if the IoU is equal to or larger than 0.5.
- AP_75: It is an AP score in the case of IoU = 0.75. A higher IoU threshold means that the predicted bounding box must overlap more area over the ground truth.
- AP_s: It is an AP score for small objects, where the area of the object is smaller than $32^2$ pixels. This metric evaluates how well the model can detect small objects.
- AP_m: It is an AP score for medium objects, where the area of the object is between $32^2$ and $96^2$ pixels. This metric evaluates how well the model can detect medium-sized objects.
- AP_l: It is an AP score for large objects, where the area of the object is larger than $96^2$ pixels. This metric evaluates how well the model can detect large objects.

### Comparisons with other methods

#### On the COCO dataset

To give a comprehensive comparison between the models, the quantitative scores of mAP, AP_50, AP_75, AP_s, AP_m and AP_l obtained by the 13 models on the COCO dataset are listed in Table 1. The evaluation metrics are presented in “Experimental settings and evaluation metrics”. As can be seen from the Faster-RCNN, which has been proved to be one of the best two-stage methods, our model achieves a large enhancement over 3.3% on the mAP score. In addition, the mAP score obtained by our model is 14.1% higher than that with the classical one-stage model SSD. By contrast to the YOLOX, from which our model is developed, we achieved about 3.3% enhancement on the mAP score. For the latest developed transformer based methods, our method even achieves about 3.7% and 2.5% enhancement over DETR and conditional DETR, and also performs a little better than UP-DETR and FP-DETR on the mAP score. As for the more strict score AP_75, our method renders greater superiority with 3.7%, 16.8%, 4.3% higher over the Faster-RCNN, SSD, and YOLOX, and even outperforms the transformer based methods. This fact indicates that our model is an effective yet useful object detector.

**Table 1:** Quantitative comparison results on the COCO testing set.

Methods	mAP	AP_50	AP_75	AP_s	AP_m	AP_l
Faster-RCNN^12^	40.3	61.0	44.0	24.0	44.1	51.4
Cascade-RCNN^48^	41.0	59.4	44.4	22.7	44.4	54.3
RetinaNet^22^	37.4	56.7	39.6	20.0	40.7	49.7
SSD^20^	29.5	49.3	30.9	12.1	34.1	44.9
PP-YOLO^34^	39.3	59.3	42.7	16.7	41.4	57.8
YOLO v3^29^	33.7	56.6	35.3	19.4	36.8	44.3
YOLO v5^31^	37.4	57.0	40.9	20.9	42.5	48.8
YOLO v7^33^	38.7	56.7	41.7	18.8	42.4	51.9
YOLOX^45^	40.3	59.1	43.4	23.5	44.5	53.1
DETR^38^	39.9	60.4	41.7	17.6	43.4	59.4
Conditional DETR^40^	41.1	61.9	43.5	20.4	44.5	59.9
UP-DETR^41^	43.1	63.4	46.0	21.6	46.8	62.4
FP-DETR^42^	43.2	63.1	47.5	25.7	46.7	57.5
Our method	43.6	62.0	47.7	26.7	47.5	58.0

As the COCO dataset is a challenging benchmark for object detection, in which there are many classes of objects with different sizes, we divided the scores into AP_s, AP_m and AP_l to evaluate more finely the performances of the 13 models on the small, medium and large objects, respectively. It is seen that our model achieves the best performance on these three scores compared with the 13 models, except that the AP_l score is a little lower than the transformer based methods, DETR, Conditional DETR, and UP-DETR. Actually, the design of our model is motivated from the principal of human vision perception by comparing together all of the objects with different sizes as a whole. Computationally, the multi-scale coupled attention is developed to reach this goal. In this process, multi-scale feature maps are merged together with self-attention learning from each other. The comparative results indicate the effectiveness of the MSCA network.

#### On the KITTI sataset

Note that the images in KITTI dataset are taken at the automatic driving scenarios. By contrast to the COCO dataset, objects in these images are more densely distributed, but the differences of object sizes change relatively less significantly. Following the evaluation metrics that are used in most existing works on this dataset, here we use the precision (P), recall rate (R), and AP_50 to assess the performances of the ten objects. It is worthy pointing out that for scenes with densely-distributed objects, the precision and recall rate are two fundamental yet important metrics to measure the performances of the models.

The scores of the precision, recall rate, and AP_50 are reported in Table 2. Compared by the Faster-RCNN, our model achieves a large enhancement over 3.7% on the P score, 4.7% on the R score. By contrast to the SSD method, the P and R scores are increased over 6.9% and 8.7%, which renders a significant enhancement on model performance. In addition, compared with the YOLOX, our model achieves about 1.7 % enhancement on the P score and 2.7% on the R score. On the AP_50, compared with the nine models, our model also achieves the best result. This fact validates the effectiveness of our model.

**Table 2:** Quantitative comparison results on the KITTI testing set.

Methods	P	R	AP_50
Faster R-CNN^12^	89.1	92.8	91.9
Cascade R-CNN^48^	88.5	91.9	91.2
RetinaNet^22^	85.2	88.7	87.2
SSD^20^	85.9	88.8	87.5
PP-YOLO^34^	–	–	86.9
YOLO v3^29^	89.2	90.9	90.8
YOLO v5^31^	89.7	93.5	92.9
YOLO v7^33^	92.3	97.1	95.2
YOLOX^45^	90.5	94.8	94.0
Our method	92.8	97.5	96.0

### Ablation study

#### Overview of the ablation study

Note that, architecturally, in our work there are a few fundamental designs. This subsection reports the extensive ablation experiments to evaluate the importance of the proposed model in our method with different configurations. Without loss of generality, we employ the COCO dataset to conduct the experiment, which is relatively more larger than the KITTI benchmark. For clarity, the ablation study conducted in the following aspects:

- Model component evaluation. The key model component designed in this work is the proposed MSCA network. As demonstrated in Fig. 1, it contains a sequence of the MSCCA and MSCSA modules with totally *L* length. The performances of the models with or without them will be evaluated. In addition, the models with different sequence lengths of modules MSCCA-MSCSA will be also assessed for guiding the practical usage. Furthermore, as demonstrated in Fig. 2, the MSCA network takes the mixture of the three-scales of features as its input. Naturally, such a mixture could not be jointly performed. That is, the MSCA can be employed individually for each scale of feature maps. Such a topology change will also be evaluated experimentally.
- The effect of the NonLM. In “The MSCSA”, we introduce the Non-Linear Mapping (NonLM) function formulated in (12), which acts actually as an activation function to map the features into a latent space for similarity estimation between features. The simplified functions reduced from the $NonLM(\cdot )$ will be evaluated for comparisons.
- The use of the MSCA. Architecturally, acting as the neck network, the MSCA is attached after the PAFPN module. As demonstrated in Figs. 1 and 2, it outputs three scales of feature maps. Naturally, MSCA network may be applied individually to a scale feature map or simultaneously to different scales of feature maps. Thus, we have conducted the ablation study on the use of the MSCA to illustrate its effect on the performance of the model.

The details about the ablation studies are given in the next subsection, in which some explanations are made for clarity.

#### Model component evaluation

To validate the effectiveness of our proposed models, experiments are designed for component evaluation, where the MSCCA, MSCSA, and the NonLM modules are attached gradually to estimate their contribution to the performance of the network. The experimental results are reported in Table 3. In the experiments, the sequence length of MSCAs, namely the structure parameter *L* in Fig. 1, is set to be 2. In Table 3, the “Feature Concat” stands for the operation of mixing together all of the multi-scale features. It associates to the operation “$\oplus$” in Fig. 1, and the details about the dataflow are also illustrated in Fig. 2. To demonstrate the advantages of our network structure objectively, the evaluation metrics, including the mAP, AP_50, AP_75, AP_s, AP_m, and AP_l, are reported for comparisons.

**Table 3:** Component evaluation experiments on the modules of MSCCA, MSCSA and NonLM. The experimental dataset is COCO and the baseline is YOLOX.

Methods	Coupled Attention		Feature Concat	NonLM	mAP	AP_50	AP_75	AP_s	AP_m	AP_l
	MSCCA	MSCSA								
YOLOX	$\times$	$\times$	$\times$	$\times$	40.32	59.10	43.41	23.49	44.53	53.11
Our method	$\checkmark$	$\times$	$\checkmark$	$\times$	41.39	59.81	44.92	25.43	45.68	54.43
	$\checkmark$	$\checkmark$	$\checkmark$	$\times$	42.84	61.32	46.59	26.10	47.22	56.78
	$\checkmark$	$\checkmark$	$\times$	$\checkmark$	42.51	60.73	45.91	26.02	46.74	55.68
	$\checkmark$	$\checkmark$	$\checkmark$	$\checkmark$	43.62	62.01	47.70	26.73	47.51	58.02

Note that in the case that the MSCCA, MSCSA, $\oplus$ and NonLM are all not used, our model will be reduced to the YOLOX^45^). As can be seen from Table 3, with the modules attached gradually, the performance of the model for object detection is significantly improved. For example, in the case that only the MSCCA module is used, the mAP score is enhanced more than 1.0%, compared with its baseline YOLOX. When further adding the MSCSA module with NonLM operation, our results are getting better and better. The best results are achieved when the MSCCA, MSCSA, NonLM modules are all added into the original network. In this case, our model achieves more than 3.3% enhancement on the mAP score, compared with the YOLOX. In addition, the scores of AP_50, AP_75, AP_s, AP_m, and AP_l are all significantly enhanced.

**Figure 6:**
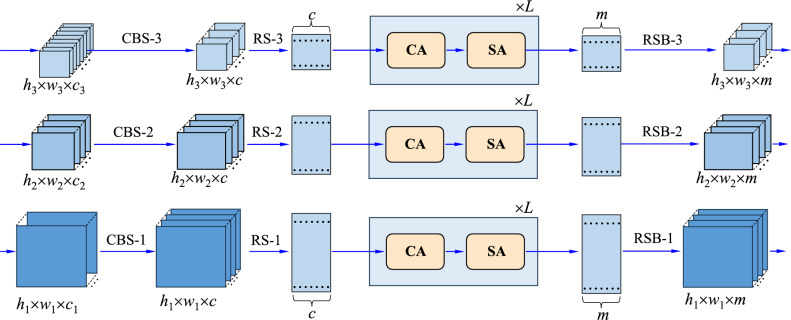
The neck structure with the MSCA added individually into each scale of feature maps. In parallel to Fig. 2, here the module “CA” has the same structure of the “MSCCA”, and the module “SA” has the same structure of the “MSCSA”.

Additionally, another effort is tried to validate weather the $\oplus$ operation for multi-scale feature mixture in our proposed MSCA can help improve the performance. To this end, a new neck network is designed by assembling the modules MSCCA and MSCSA only within each scale of feature maps. That is, the mixture operation for multi-scale features will not be performed. In parallel to Fig. 2, Fig. 6 shows the structure designed for this case. With such a structure configuration, we trained the model, and its performance is reported in Table 3 (see the penultimate row). It is seen that the use of modules MSCCA and MSCSA helps improve the performance over the original model also significantly. This further indicates their effectiveness used in the YOLO framework for object detection. More importantly, when the $\oplus$ operation for mixing together the multi-scale features is performed, the performance is clearly enhanced. This fact can be witnessed from the scores in the last two rows in Table 3. This indicates the necessity of the mixture operation for multi-scale features within the proposed MSCA network.

As demonstrated in Fig. 1, our MSCA network consists of two sub-modules: MSCCA and MSCSA. It is also illustrated that this coupled attention module can have *L* repetitions in the network structure. To assess the effect of the structure parameter *L*, another experiment has been conducted by changing *L* from 1 to 4. The experimental settings keep unchanged as the previous experiments. The results are reported in Table  4.

**Table 4:** The precision of different metrics with different sequence length (*L*) of modules {MSCA} in Fig. 1. The number is increased from 1 to 4 for comparisons.

Number (*L*)	mAP	AP_50	AP_75	AP_s	AP_m	AP_l
1	43.02	61.03	46.74	26.01	46.60	56.30
2	43.62	62.01	47.70	26.73	47.51	58.02
3	43.53	61.94	47.70	26.52	47.53	58.11
4	43.30	61.69	47.03	26.50	46.68	56.84

According to the results reported in Table 4, in the case of $L=2$, the scores of most metrics are the best on average. Some results may be better when $L=3$ ($AP\_m=47.53$ and $AP\_L=58.11$). In experiments, it has been observed that, when $L\ge 4$, the metric scores are beginning to decrease. For the above considerations and taking into account the computational cost, we suggest $L=2$ for real-world applications.

#### The effect of the NonLM

In “The MSCSA”, we explore to design a NonLM to mine more intrinsic discriminative details from the feature maps generated by the MSCSA module. Technically, the NonLM function in (12) transforms the *d*-dimensional features into a latent space for similarity estimation. It is actually a combination of the traditional ReLU and the exquisite ELU (exponential linear unit^56^), followed to the two linear mappings respectively.

To this end, experiments have been conducted to compare the NonLM, ReLU and ELU on the COCO dataset. The evaluation metrics are the mAP, AP_50, AP_75, AP_s, AP_m, and AP_l, and their scores are reported in Table 5 for comparisons. The experimental settings keep unchanged as the previous experiments with sequence length of MSCAs equal to 2. We see that the activation function of ELU has some advantages over the ReLU function. By contrast, our proposed NonLM shows better performances over both the ReLU and the ELU function.

**Table 5:** The performances on the COCO dataset by the method with different activation functions, including ReLU, ELU and our proposed NonLM.

Function	mAP	AP_50	AP_75	AP_s	AP_m	AP_l
ReLU	42.84	61.32	46.59	26.10	47.22	56.78
ELU	43.02	61.50	46.81	26.29	47.24	57.23
NonLM (Our)	43.62	62.01	47.70	26.73	47.51	58.02

#### The use of the multi-scale coupled attentions

**Figure 7:**
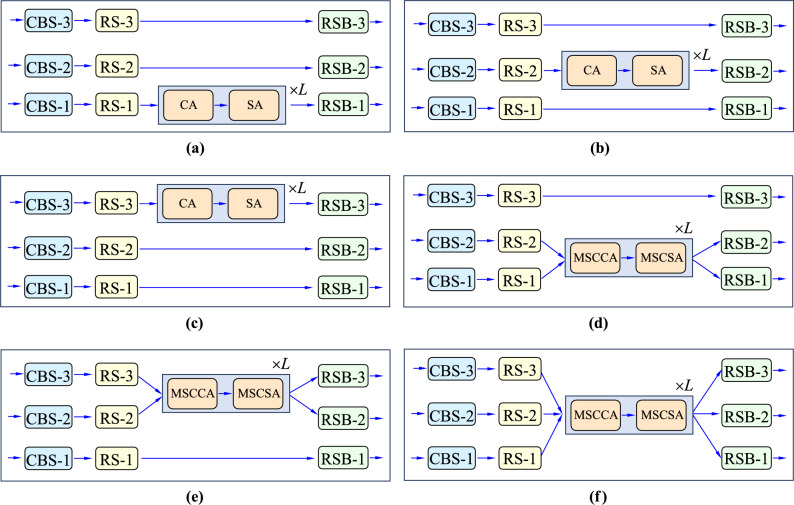
Different topological structures whether the proposed MSCA network are used or not at different scale of feature maps. In parallel to Fig. 2, here the module “CA” has the same structure of the “MSCCA”, and the module “SA” has the same structure of the “MSCSA”. (**a**) the structure using our design only at the first scale, (**b**) the structure using our design only at the second scale, (**c**) the structure using our design only at the second scale, (**d**) the structure using the MSCA (namely, MSCCA + MSCSA) simultaneously at the first and the second scales, (**e**) the structure using the MSCA simultaneously at the second and the third scales, (**f**) the structure using the MSCA simultaneously at all of the three scales. Note that the structure in (**f**) is just the one shown in Fig. 1.

In Fig. 1, the feature maps at three scales are merged together as the input of the MSCA. The cross-scale self-attention is performed by learning from all of them as a whole. In this ablation study, the contribution of the multi-scale coupled attention has been investigated. To this end, besides the model in Fig. 1 (also corresponding to that in Fig. 7f), five new modules are constructed by replacing the MSCA in Fig. 1, which are illustrated in Fig. 7a–e.

Specifically, in Fig. 7a, the model is constructed by using the “CA+SA” at the first scale. Here the module “CA” has the same structure as the “MSCCA”, and the module “SA” has the same structure as the “MSCSA”. But their inputs are only those from a single scale. With the model configuration in this work, it corresponds to the spatial resolution by down-sampling the original images 8-times. For example, for original images with 640$\times$640 pixels, the spatial size of the feature maps at this scale now turns to be 80 $\times$ 80. In Fig. 7b, the model is constructed by using the “CA+SA” at the second scale. It corresponds to the spatial resolution by down-sampling the original images 16-times. In Fig. 7c, the model is constructed by using the “CA+SA” at the third scale. It corresponds to the spatial resolution by down-sampling the original images 32-times.

In Fig. 7d, the model is constructed by using the MSCA simultaneously at the first and the second scales. In Fig. 7e, the model is constructed by using the MSCA simultaneously at the second and the third scales, and In Fig. 7f the structure using the MSCA simultaneously at all of the three scales. Note that the structure in Fig. 7f is just identical to the one shown in Fig. 1. In Fig. 7d–f, the sequence length of MSCAs is set to be 2 (namely *L* = 2). In these six models, all the other parts of the models, including the CSPDarkNet, PAFPN and the output layer, are kept as the same as those in the YOLOX. In addition, The experimental settings keep unchanged as the previous experiments.

The results obtained from these six models are presented in Table 6. It is observed that, when there is only one scale that uses the of MSCA, the results are less better. When the number of scales increases to two, the results get better. Typically, when the coupled attentions are mutually performed on the scales (16, 32) (namely, the second and the third scales in Fig. 7e), some results are even best for the precision of mAP, AP_50, AP_s and AP_m. However, on average, best results are obtained when all of the three scale features are merged together for MSCA. This fact indicates that our design of the multi-scale coupled attentions is effective for improving object detection.

**Table 6:** The performances of the six models constructed in Fig. 7, which are conducted on the COCO dataset.

Model	Scale using the MSCA	mAP	AP_50	AP_75	AP_s	AP_m	AP_l
Figure 7a	8	42.02	60.10	45.61	25.87	45.84	55.13
Figure 7b	16	42.11	60.37	45.63	25.82	45.99	55.27
Figure 7c	32	41.84	59.70	45.68	25.21	45.93	55.24
Figure 7d	(8, 16)	43.23	61.63	46.67	26.51	47.49	57.04
Figure 7e	(16, 32)	43.56	61.98	47.68	26.44	47.51	57.70
Figure 7f (=Figure 1)	(8, 16, 32)	43.62	62.01	47.70	26.73	47.51	58.02

### The performance behavior of our model

Note that our model is developed on the YOLOX framework, in which the MSCA is used as its neck component. As demonstrated in the comparisons against the 13 widely-used models in the field of computer vision and the extensive ablation studies conducted in this work, the MSCA helps improve the performance of object detection significantly.

**Figure 8:**
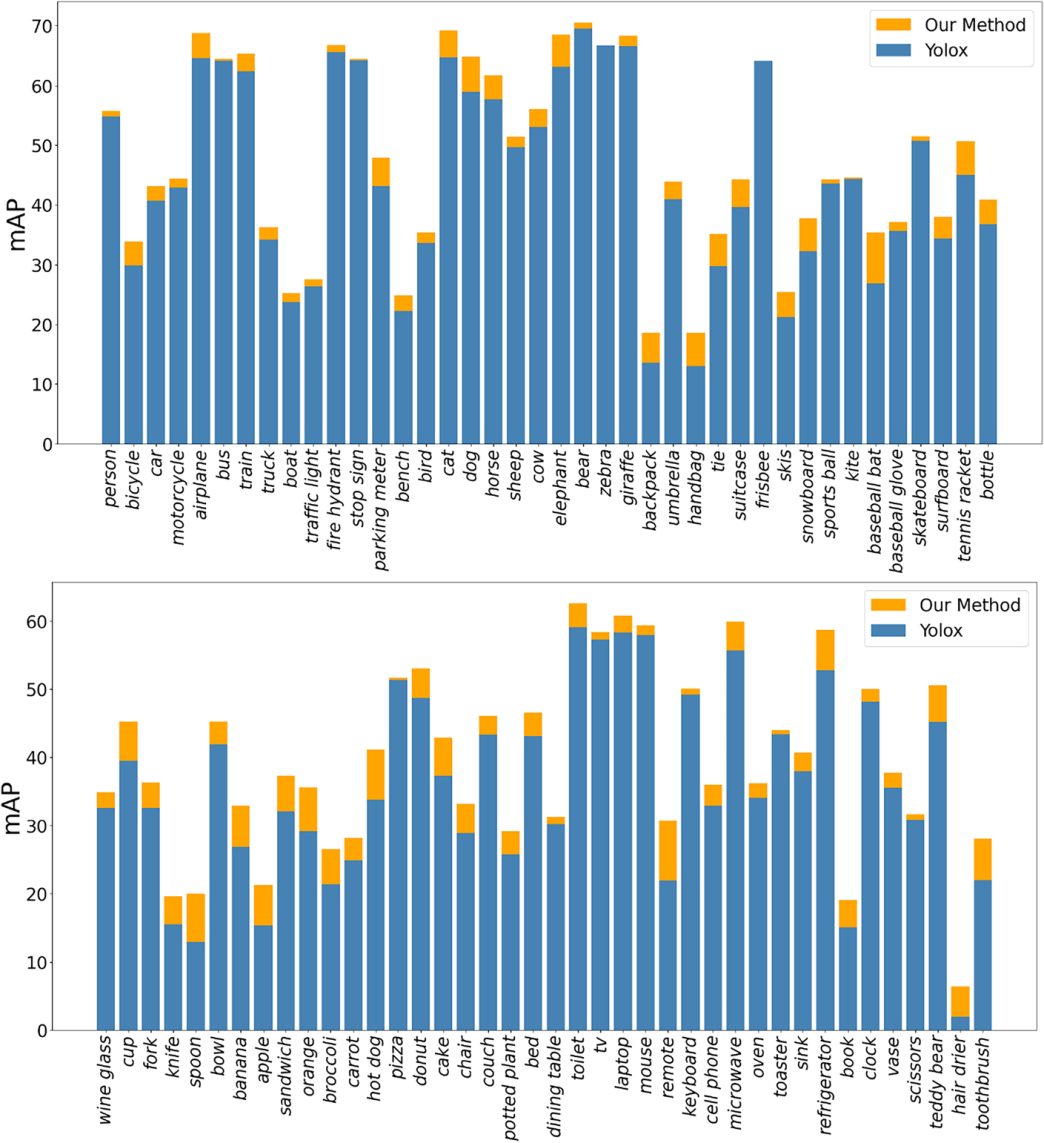
The mAP of each class on the COCO dataset.

In this subsection, we further investigate the performance behavior by taking the YOLOX as the baseline. Figure 8 demonstrates the mAP scores of the eighty categories obtained by our model and the YOLOX, which are conducted on the COCO dataset with the experimental setting described in “Experimental settings and evaluation metrics”. These eighty categories are composed of differen sizes of objects. It is seen that our model achieves higher mAP score on each of the eighty categories. Thus, it indeed improves the performance of the YOLOX by adding the MSCA.

**Figure 9:**
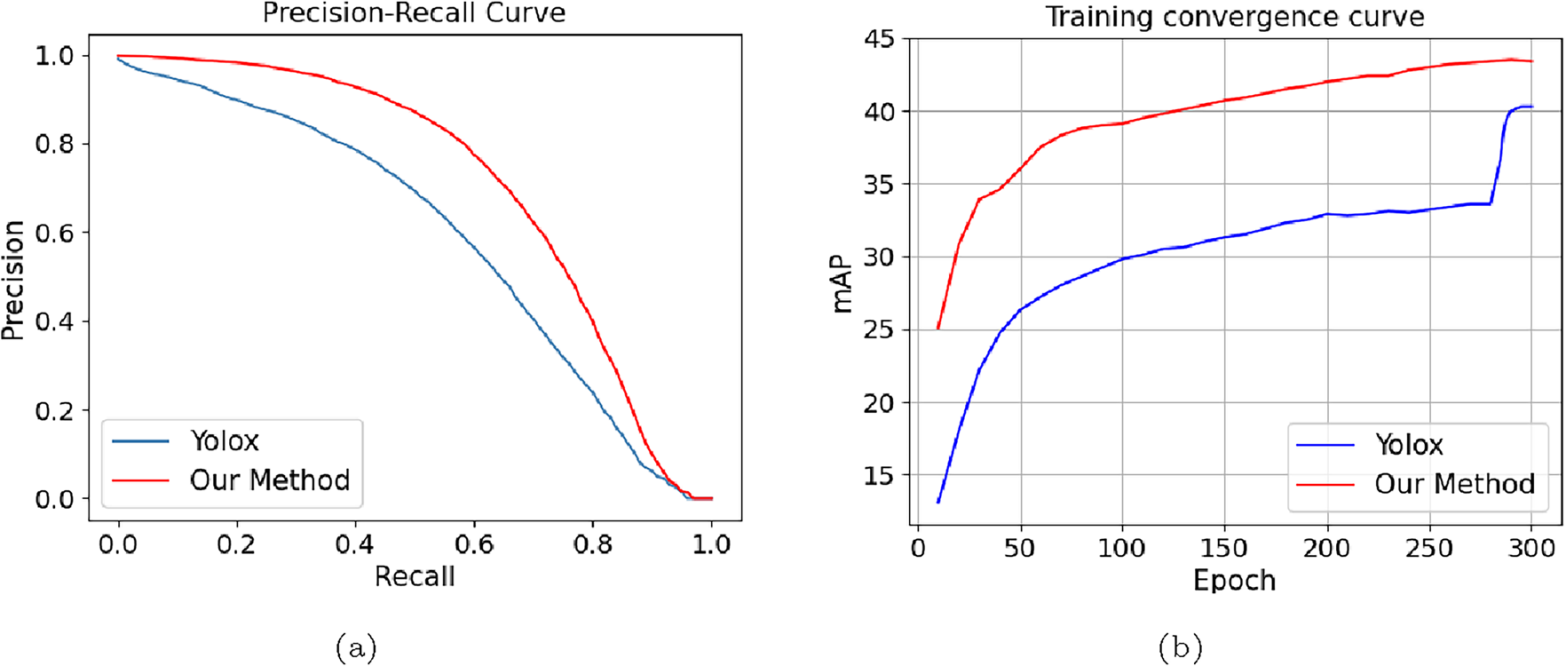
(**a**) The Precision-Recall curves obtained by our model and the YOLOX; (**b**) The training convergence curves of our model and the YOLOX. The experiments are conducted on the COCO dataset.

Furthermore, Fig. 9 illustrates the Precision-Recall (PR) curves and the training convergence curves of our model and the YOLOX. It can be observed from Fig. 9a that the PR curve obtained by our model is always above that obtained by the YOLOX. In addition, as demonstrated in Fig. 9b, the mAP scores obtained by our model at the training epochs are stably higher than those of the YOLOX. It can be also seen that near the 300-th epoch, the mAP curve tends to be stable, without large increase, indicating it arrives near the convergence point. The above observations demonstrates the fact that the design of the MSCA can help improve of the performance in training.

**Figure 10:**
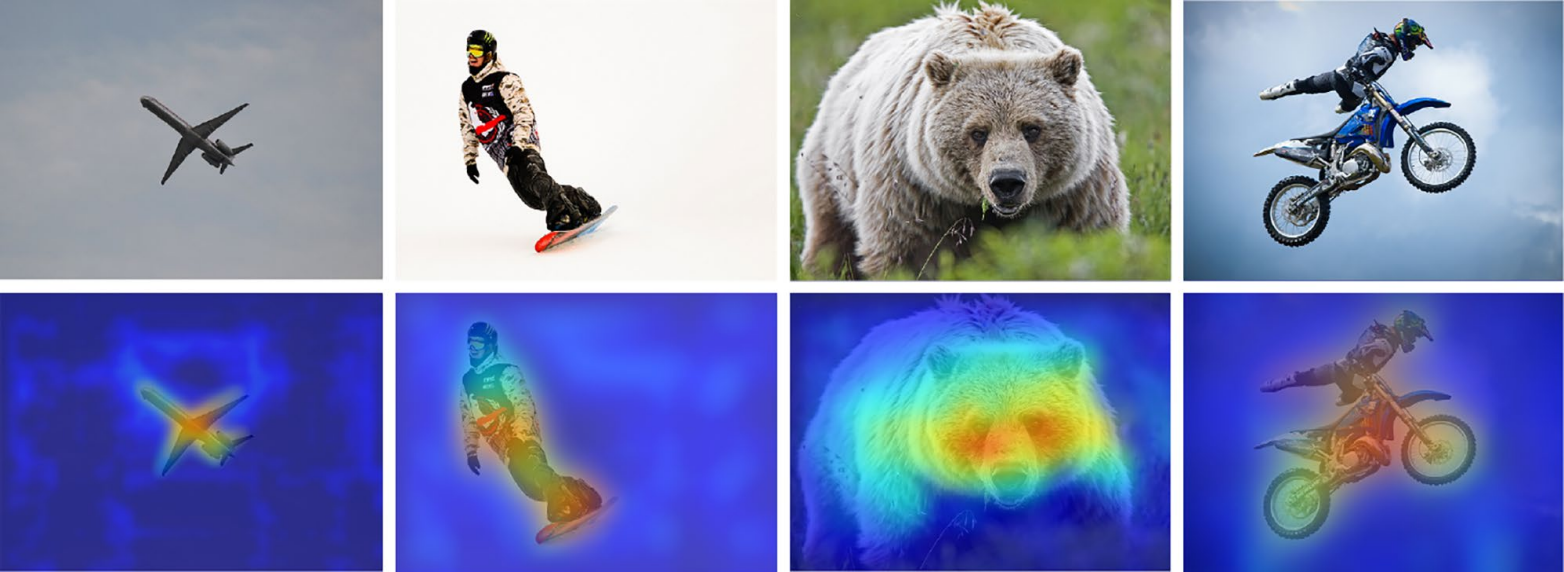
Four examples of visualization on the spatial attention generated by the final MSCSA module. The image are taken from the COCO dataset.

Finally, Fig. 10 demonstrates the sensitivity of our MSCA to some of the interested target objects. Actually, it is the importance of the cross-scale self-attention on the spatial gird, which is obtained at the final layer of the MSCSA. The figure is obtained via the following steps. First, based on the final similarity $sim\left( {{\textbf{q}}_i,{\textbf{k}}_j } \right)$ formulated in Eq. (13), for each feature point *i*, the sum ${\textbf{I}}_{i}$ is obtained by adding the $sim\left( {{\textbf{q}}_i ,{\textbf{k}}_j } \right)$ on all *j* together. That is, ${\textbf{I}}_{i}={\sum }_{j}sim\left( {{\textbf{q}}_i ,{\textbf{k}}_j } \right)$. This sum ${\textbf{I}}_{i}$ is then taken as the sensitivity at feature point *i*. Second, since the feature points are taken at three different scales, we only pick out the first $s_1$ sums at the first scale and reshape them back to a matrix. That is, the self-attention sensitivity only on the first scale is visualized. In other word, now it is a $80\times 80$ matrix. Finally, it is up-sampled with super-resolution tricks to obtain the visualization result with the same as the input image. From the examples demonstrated in Fig. 10, it can be observed that our method really captures the important regions for object detection.

## Conclusions

This paper has proposed a Multi-Scale Coupled Attention (MSCA) network for object detection. In this model, the core unit in MSCA is divided into two attention operations: the Multi-Scale Coupled Channel Attention (MSCCA), and the Multi-Scale Coupled Spatial Attention (MSCSA). Architecturally, these two attention operations are bundled together, and can be repeated several times. Both of them are construed on the mixture of multi-scale features, which are taken equally as a whole for self-attention learning. Typically, the MSCCA focuses on how to develop the linear attention on the channels that are employed to represent the visual features of objects. In parallel, MSCSA lays emphasis on how to construct the non-linear attention on the spatial grid by comparing the multi-scale features together against each other. Topologically, the MSCA network is designed as plugin module, which can be used to learning mutually from cross-scale features or individually from single scale features. The usability of the proposed MSCA network has been evaluated via the comparisons against the powerful models that are widely used in industrial applications. Its usability has also been demonstrated via the ablation studies with a series of model model variants, and the analyses on the performance behaviour. The extensive comparative experiments indicate that the MSCCA network helps improve significantly the performance of the models in the YOLOX framework.

## Data Availability

The datasets used and/or analysed during the current study available from the corresponding author on reasonable request.
